# A Web-Based Gender-Sensitive Educational Simulation on Vocational Rehabilitation for Service Providers Working With Youth With Disabilities: Pilot Evaluation

**DOI:** 10.2196/38540

**Published:** 2023-03-24

**Authors:** Sally Lindsay, Nicole Thomson, Sandra Moll, Angela Colantonio, Jennifer Stinson

**Affiliations:** 1 Holland Bloorview Kids Rehabilitation Hospital Toronto, ON Canada; 2 University of Toronto Toronto, ON Canada; 3 McMaster University Hamilton, ON Canada; 4 Hospital for Sick Children Toronto, ON Canada

**Keywords:** continuing professional development, CME, medical education, professional development, continuing education, gender-sensitive care, online, gender, education, simulator, simulation, clinician, pilot, youth, young, disabled, disability, child, pediatric, disabilities, trainee, community, training, tool, rehabilitation, feedback, survey

## Abstract

**Background:**

Although there is a need for gender-specific health care, especially within the context of vocational rehabilitation for youth with disabilities, clinicians, trainees, and community service providers commonly report lacking training in gender-sensitive approaches. Therefore, an educational tool designed for clinicians working with youth, that addresses how to approach such issues, could help clinicians to augment the care they provide.

**Objective:**

The objective of our study was to conduct a pilot evaluation of an educational simulation for health care and service providers focusing on gender-sensitive approaches within the context of supporting youth with disabilities in vocational rehabilitation.

**Methods:**

We conducted a survey from May to September 2021 to assess the relevance of the simulation content, preliminary perceived impact on gender-sensitive knowledge and confidence, and open-ended feedback of a web-based gender-sensitive educational simulation. A total of 12 health care providers from a variety of professions who had experience working with youth in the context of vocational rehabilitation participated in the survey (11 women and 1 man).

**Results:**

Most participants reported that the content of the simulation was relevant and comprehensive. The majority of participants reported that the simulation helped to increase their perceived knowledge or understanding of the topic, changed their perceived understanding of their intervention or approach, and informed their perceived confidence. Our qualitative findings from the open-ended questions highlighted three main themes: (1) relevance of the simulation content, (2) perceived impact for clinical practice (ie, gender-sensitive language and communication and building rapport with patients), and (3) perceived impact on organizational processes (ie, practices, policy, and privacy).

**Conclusions:**

Our educational simulation shows preliminary potential as an educational tool for service providers working with youth who have a disability within the context of vocational rehabilitation. Further research is needed to assess the impact of the tool with larger samples.

## Introduction

### Background

The World Health Organization advocates for a broad approach to how sex and gender issues are addressed both within and outside of the health care sector [1]. Gender is an important social determinant of health and refers to socially constructed roles, behaviors, expectations, and identities that vary with roles, norms, and values within society [1,2]. Given the importance of gender on influencing health behaviors and health outcomes, it is critical that health care providers are equipped to offer appropriate care that addresses the needs of their patients [3]. Gender-sensitive care is aligned with patient- and family-centered care approaches as it focuses on interacting with patients in a respectful manner, working in a partnership, and sharing information to make informed choices [4-6]. Gender-sensitive approaches involve understanding the gendered health care patterns (including norms, stereotypes, and inequalities) of men, women, and nonbinary or gender-fluid individuals [2,7,8], while also reflecting on the wider cultural and sociopolitical context of health care environments [3,7]. Although health care providers increasingly recognize the significance of gender within their clinical practice, there are very few gender-sensitive training tools available, and many health care providers report lacking training in this area [9,10]. Therefore, addressing this gap is crucial because clinicians’ attitudes toward gender could affect their behaviors and potentially bias their assessments of patients, impacting the quality and equity of care [3,11,12]. Despite the increased attention to gender-sensitive approaches, most studies concentrate on adult populations with little attention to youth.

In this paper, our educational tool focuses on providing gender-sensitive care to youth and young adults because their gendered experiences are often ignored or dismissed despite gender playing a vital role in their health and social outcomes [13-15]. In particular, youth who identify as lesbian, gay, bisexual, trans, or queer two-spirited (LGBTQ2Si+) have often been overlooked within rehabilitation settings [3,10,16]. A recent study from our team focusing on the challenges of providing gender-sensitive care within a pediatric rehabilitation hospital highlighted that clinicians often encountered obstacles regarding the complexity of patients’ gender identity and especially how to communicate with patients identifying as nonbinary or trans [17]. Lack of training in working with such patients is concerning for clinicians because trans and nonbinary youth often experience barriers within health and social services (ie, stigma and discrimination) and are also at higher risk of mental health conditions such as depression and suicide [18]. Therefore, it is important that clinicians receive appropriate training in how to communicate effectively with patients based on their gender identity.

### Gender-Sensitive Care Within the Context of Vocational Rehabilitation for Youth With Disabilities

In this study, our educational simulation focuses on gender-sensitive approaches within the context of vocational rehabilitation to youth with disabilities. This topic is important because gender often influences how people with disabilities are engaged within vocational training and employment [10,19]. For instance, a systematic review focusing on the role of gender in employment among youth with disabilities reported that young women with disabilities often lag behind men with disabilities on several health and social outcomes, including lower employment rates and multiple forms of discrimination [10,19,20]. Some research indicates that women vocational rehabilitation consumers are more likely to be placed with a lower starting wage and receive less job readiness training, on-the-job training, job search assistance, and job placement assistance than men [8]. Additionally, young women with disabilities often experience specific challenges with career development including disability stereotypes, lower family expectations, decreased self-confidence, and limited vocational training [21,22].

Although several studies highlight a critical need for gender-specific vocational support for youth with disabilities [23-25], relatively little attention has been given to gender within the context of disability and employment [20,26-28]. The few studies exploring gender and employment among youth with disabilities note differences in employment outcomes or pay [10,19]. A key limitation in previous research is that most studies explore gender from a binary (ie, man or woman) perspective and do not include diverse perspectives of gendered experiences. Therefore, enhancing our understanding of gender, within the example of providing vocational rehabilitation for youth with disabilities, is salient for decision-making, enhanced communication, and engagement in programs and interventions [29]. Our study developed a gender-sensitive educational simulation within the context of vocational rehabilitation to explore this issue.

## Methods

### Objective

The objective of our study was to conduct a pilot evaluation of an educational simulation on gender-sensitive approach within the context of vocational rehabilitation for youth with disabilities.

### Design and Procedure

We conducted a survey to assess the relevance and initial feedback of our web-based simulation educational tool with the intention to examine the methods and procedures to test at a larger scale [30]. After consenting to participate, clinicians were asked to view the simulation (approximately 10 minutes) remotely, on their own, via a password-protected portal. Given that the data collection took place during the COVID-19 pandemic with public health restrictions for in-person meetings, we were unable to conduct an in-person focus group discussion alongside the surveys. After viewing the simulation, participants completed a survey that a researcher sent via a Research Electronic Data Capture (REDCap) link. Study data were collected and managed using REDCap, a secure web-based software platform designed to support data capture for research studies hosted at our institution [31].

### Ethics Approval

This study received institutional research ethics board approval from Holland Bloorview Kids Rehabilitation Hospital and University of Toronto (#0162). All participants provided written consent (or e-consent via REDCap during the pandemic) before taking part in the study [31]. Participants who completed the survey received a CAD $20 (US $14.47) gift card as a token of appreciation for their time, as recommended by our research ethics board. Data were anonymized and deidentified before analysis to protect the privacy and confidentiality of participants.

### Development of the Educational Simulation

Our intervention involved an educational simulation, which is an interactive pedagogy allowing clinicians and service providers to practice assessment, patient care, and difficult conversations in a risk-free environment [32]. Educational simulations are considered a gold standard for skill development and learning and can be a valuable pedagogical tool for interprofessional training, while having the potential to improve patient outcomes [33-36]. Our educational simulation (consisting of a 10-minute web-based video) was targeted for a variety of vocational rehabilitation clinicians and service providers (eg, occupational therapists, social workers, employment counselors, and related staff) who support youth with disabilities in finding and maintaining employment [37]. The educational tool addressed gender-sensitive approaches to engage patients within vocational rehabilitation (eg, rapport development, gender-role expectations, self-advocacy, disclosure decisions, and career development) and self-reflection on the role of gender within clinical practice. Various gendered perspectives were showcased while drawing on evidence-informed lived experiences of working with youth with disabilities. The simulation was co-created with clinicians (eg, occupational therapists, social workers, and employment counselors) who work with youth with disabilities (see [37] for full description of the simulation development). During the co-creation of this tool, clinicians and youth with disabilities decided to have the simulation focus on youth with acquired brain injury, a condition commonly seen at our hospital. The simulation involved creating a scripted scenario, which was portrayed by actors (ie, standardized patients). The rationale and content of the simulation were based on systematic reviews conducted by our team focusing on the role of gender and employment among youth with disabilities [19] and need assessments with clinicians and youth with disabilities [10,38]. The simulation was designed to be delivered as a continuing education tool to clinicians and service providers (eg, occupational therapists, social workers, youth employment counselors, and youth facilitators) and also to trainees currently working in the area of supporting youth with disabilities in vocational rehabilitation especially in regard to finding and securing employment. The tool can be requested and accessed at the URL in [39].

### Sample and Recruitment

Data were collected from May to September 2021 and led by the first author with support from a research assistant. Using a purposive sampling strategy, aiming to recruit approximately 10 clinicians in vocational rehabilitation working with various types of patients with disabilities, participants were recruited through invitation letters and advertisements at a pediatric rehabilitation hospital and community-based disability-related organizations and relevant social media platforms. This sample size is considered appropriate for a pilot study to assess the usability of a web-based tool [40]. We screened participants for inclusion with the following eligibility criteria: a health care provider, practitioner or community service provider, or trainee who had relevant experience in helping young people with disabilities to find employment (eg, occupational therapist, job coach or counselor, social worker, etc).

A total of 12 participants (11 self-identified as women and 1 man) took part in the study (see Table 1 for overview). It is important to note that 10 participants had a complete survey, whereas 2 had some missing data. Participants ranged in age from 20 to 60 years, with the majority of them aged 26-40 years. They had practiced as a clinician in this field from less than 1 year to over 20 years, with the majority practicing between 3 and 10 years. Most participants (9/12, 75%) had a master’s degree or higher level of education.

**Table 1:** Overview of participant characteristics.

Participant	Profession^a^	Gender	Highest level of education	Number of years practicing	Nijmegen Gender Awareness score (mean 13.75)
1	Occupational therapy assistant	Woman	College certificate	3-5	14
2	Youth facilitator^b^	Woman	Bachelor’s degree	1-2	12
3	Social worker	Woman	Master’s degree	20	13
4	Youth facilitator (disability employment support)^b^	Man	Bachelor’s degree	6-10	17
5	Occupational therapist	Woman	PhD	1-2	17
6	Occupational therapist	Woman	Master’s degree	3-5	14
7	Social worker	Woman	Master’s degree	3-5	9
8	Occupational therapist	Woman	Master’s degree	6-10	14
9	Occupational therapist	Woman	Master’s degree	<1	13
10	Occupational therapist	Woman	Master’s degree	11-15	13
11	Occupational therapist	Woman	Master’s degree	<1	16
12	Social worker	Woman	Master’s degree	<1	13

^a^Involved with supporting youth in vocational rehabilitation.

^b^The youth facilitator role involves an individual with lived experience of a child-onset disability, chronic illness, or acquired injury who works as part of the rehabilitation team. They have not been an active client for at least 2 years in the service or team they are working on.

### Outcome Measures

Our primary outcomes to assess the simulation included relevance of the content and perceived impact on knowledge and confidence related to gender-sensitive care. Questions were adapted from the Community Impacts of Research-Oriented Partnerships (personal knowledge development subscale) and asked about whether participants increased or changed their personal understanding about the topic (eg, exposed to different areas of expertise and new knowledge about current research and thinking in the field and raised awareness of different issues), whether they changed their beliefs and understandings with respect to an intervention or approach or group of people (eg, led to a new way of thinking or to a broader or new perspective and altered ideas about how to best deliver service or programs), and whether it increased their confidence in their professional or daily practice or day-to-day activities [41]. These questions were measured on a 7-point scale (ie, not at all, a very small extent, to a small extent, to a moderate extent, to a fairly great extent, to a great extent, and to a very great extent) [41].

In regard to relevance of the simulation content we also used an adapted question from Malik et al’s [42] toolkit evaluation questionnaire that asked about the comprehensiveness (ie, extent to which the simulation showed rapport building and creating a safe environment) and relevance (ie, extent to which simulation showed clinician making gender-based assumptions but building rapport by reflecting on their own gender bias) of the information presented in the educational tool (ie, strongly disagree, disagree, neither, agree, and strongly agree).

To assess baseline gender sensitivity we used the Nijmegen Gender Awareness in Medicine Scale (ie, gender-sensitive subscale), which is a reliable and valid measure (ie, Cronbach α=.76, gender-sensitive scale, 0.89). This measure contains statements answered on a 5-point Likert scale (1=*totally disagree* to 5=*totally agree*) [43,44]. It measures 3 dimensions of gender awareness: gender sensitivity, gender role ideology toward patients, and gender role ideology toward doctors [43]. Here we used the subscale for gender sensitivity where a high score on the gender-sensitive subscale implies greater gender sensitivity.

Other secondary measures included demographic characteristics (ie, age range, gender, highest level of education, and years in practice). Open-ended questions involved what they liked most and least about the simulation and any suggestions for further development.

### Data Analysis

Data were anonymized and exported from REDCap into SPSS, version 25 (IBM Corp), for analysis. Descriptive statistics were used to describe sample characteristics and frequencies for categorical variables.

The qualitative data from the open-ended questions were analyzed using a directed content analysis approach [45]. Two researchers independently reviewed all of the responses to the open-ended questions and developed codes using an open-coding approach [46]. We developed a list of preliminary codes while noting patterns between them and then met to compare and contrast the codes. Team discussions helped to resolve any discrepancies in the organization of the codes and development of the themes. The first author, with experience in qualitative research, applied the final coding scheme to all of the qualitative data. Relevant quotes that reflected each theme and subtheme were extracted. Strategies to enhance rigor and trustworthiness of the findings included descriptive participant accounts and peer debriefing discussions among the research team who have expertise in rehabilitation, youth with disabilities, pediatrics, and gender [47]. We kept notes on key decisions made throughout the data analysis. The research team also reflected on their own biases and assumptions and how this may have influenced their selection of the themes, which we noted in our audit trail [47].

## Results

### Quantitative Findings

The baseline level of gender sensitivity ranged from 9 to 17 with a mean of 13.75 (see Table 1), indicating that the majority of participants had a good knowledge of gender-sensitive care. Table 2 highlights the preliminary perceived impact of the educational simulation. The majority of the participants reported a perceived increase or change in their personal knowledge or understanding of the topic (2/10, 20% to a very great extent; 4/10, 40% to a great extent; 1/10, 10% to a moderate extent; and 2/10, 20% to a small extent). Only one participant said that their knowledge did not change, which they explained was because they were already very knowledgeable about the topic.

The majority of participants (10/11, 91%) reported that the simulation changed their belief and understanding regarding their intervention or approach (2/11, 18% to a very great extent; 3/11, 27% to a great extent; 2/11, 18% to a moderate extent; and 3/11, 27% to a small extent). Most participants stated that the simulation helped to increase their perceived confidence in their professional daily practice or activities (2/11, 18% to a very great extent; 5/11, 45% to a great extent; and 3/11, 27% to a small extent).

On examining the extent to which the simulation showed rapport building and development of a safe environment, the majority of participants agreed (4/11, 36% totally agree and 5/11, 45% somewhat agree) that the tool was comprehensive (see Table 3). Additionally, 70% (7/10) of participants also agreed (4/10, 40% somewhat agree and 3/10, 30% totally agree) that the simulation was relevant in terms of portraying clinicians’ gender-based assumptions related to youths’ appearance with an ability to recover from this and to build rapport (see Table 4).

**Table 2:** Preliminary perceived impact of the simulation (N=12).

Preliminary perceived impact			Value, n (%)^a^
**Increased or changed personal knowledge or understanding of the topic (n=10)^b^**			
	Not at all	1 (10)	
	To a small extent	2 (20)	
	To a moderate extent	1 (10)	
	To a great extent	4 (40)	
	To a very great extent	2 (20)	
**Changed belief and understanding regarding intervention or approach, or topic or a group of people (n=11)^b^**			
	Not at all	1 (9)	
	To a small extent	3 (27)	
	To a moderate extent	2 (18)	
	To a great extent	3 (27)	
	To a very great extent	2 (18)	
**Increased your confidence in your professional or daily practice or activities (n=11)^b^**			
	Not at all	1 (9)	
	To a small extent	3 (27)	
	To a great extent	5 (45)	
	To a very great extent	2 (12)	

^a^Because of rounding, percentages may not total 100.

^b^Does not total to 12 due to missing data.

**Table 3:** Extent to which the simulation showed rapport building by creating a safe environment (comprehensive; n=10)^a^.

Level of agreement	Value, n (%)^b^
Totally disagree	1 (9)
Neutral	1 (9)
Somewhat agree	5 (45)
Totally agree	4 (36)

^a^Does not total to 12 due to missing data.

^b^Because of rounding, percentages may not total 100.

**Table 4:** Extent to which the simulation showed clinician making assumptions about gender based on youths’ appearance but built rapport by reflection on their own gender bias (relevance; n=10)^a^.

Level of agreement	Value, n (%)^b^
Neutral	3 (30)
Somewhat agree	4 (40)
Totally agree	3 (30)

^a^Does not total to 12 due to missing data.

^b^Because of rounding, percentages may not total 100.

### Overview of Qualitative Findings

Our findings highlighted three main themes: (1) relevance of the simulation content (relatable material and suggestions for improvement), (2) perceived impact for clinical practice (ie, gender-sensitive language and communication, and building rapport with patients), and (3) perceived impact on organizational processes (ie, gender-sensitive practices, policies, and privacy; see Multimedia Appendix 1 for an overview and exemplar quotes).

#### Relevance of Simulation Content

Most participants described the relevance of the content and format of the simulation, and the subthemes included how relatable it was and suggestions for improvement.

##### Relatable Material

Clinicians appreciated how the content could be translated into their own practice as well as for future students. For example, a participant shared:

The simulation was well-done and realistic. In my experience, I have witnessed clinicians misgender people and it is difficult to build connection or rapport again once this has occurred.

#9, Occupational Therapist

Another clinician also expressed how they felt about the authenticity of the content:

The simulation went well. The case worker and client enacted a scene that would not be unlike the ‘real world’…They stopped, reflected, apologized, asked if ok to continue. These are great steps to gain back trust and openness in a client. The smiles and body language were quite spot-on.

#4, Youth Facilitator

Others valued the relevance of the content for those with limited experience with disability disclosure, especially how it related to the role of gender in supporting youths’ employment. For example, a social worker described how pertinent the simulation content was and expressed:

It became very obvious that the clinician had mis-gendered the client. They did a great job of repairing this rupture and was honest that she had made a mistake and that it was okay as the client was happy with the interaction in the end and it appears that they felt seen and heard.

#7, Social Worker

In regard to the timing and format of the educational simulation, most participants commented on its appropriateness. For example,

With the video simulation, there was a lot of useful/helpful information shared in such a short space of time.

#5, Occupational Therapist

##### Suggestions for Improvement

A few clinicians provided some suggestions for further development, which included adding more links to other websites (eg, on gender identity, language and pronouns, and disability disclosure in employment) alongside the simulation. For example, a clinician shared, “service providers could benefit from having resources on how to support youth through the disclosure of disability process,” particularly within the context of the role that gender plays in employment (#4, Youth Facilitator).

#### Perceived Impact for Clinical Practice

The second theme focused on clinicians’ perceived impact of the educational simulation on their clinical practice, which involved the following subthemes: gender-sensitive language and communication and building rapport with patients. For example, clinicians described,

The simulations gave me the opportunity to reflect…It resonated with me and will impact my practice.

#2, Youth Facilitator

Additionally, the educational tool allowed health care providers to consider how they might incorporate these learnings. To illustrate, a social worker said,

I look forward to using the information gained from the video in my clinical practice.

#12, Social Worker

##### Gender-Sensitive Language and Communication

A subtheme of the perceived impact of the simulation on clinical practice involved gender-sensitive language and communication. Clinicians described how this educational tool helped them to consider the language they use. To illustrate, an occupational therapist said:

This tool will allow me to reflect on the language I use with clients. It has prompted me to reflect on my interactions with clients and avoid using any biased language or making assumptions.

#9, Occupational Therapist

Others agreed that watching the interactions in the simulation was helpful for considering how they could incorporate it into their practice and responding to difficult topics.

Some participants described how they learned about the importance of gender-sensitive language and being respectful of gender identity. An occupational therapist described:

I appreciated that the clinician asked the patient to let her know if she makes a mistake with their pronouns, who they would like to have access to the information about their pronouns (whether it's family or future employers), and about finding a place where they can fit in where their interests and whole person are really respected

#6, Occupational Therapist

In using gender-sensitive language and communication, clinicians described how the simulation led them to reflect on their own gender identity while making an effort to reduce gender-based assumptions.

##### Building Rapport With Patients

Another subtheme, aligned with gender-sensitive language and communication, included building rapport with patients. Participants explained how the simulation was valuable for informing their thinking about using appropriate language and awareness of gender identity and any gender-based biases or assumptions to build rapport with their patients. For example, an occupational therapist described,

I appreciated how much useful information was shared with regards to how to build rapport after reflecting on one's own gender bias and how to make sure the focus is on what is important to the patient.

#5, Occupational Therapist

In trying to build rapport with a patient, clinicians explained the usefulness of the simulation for learning the importance of being upfront in admitting any mistakes when misgendering a patient.

#### Perceived Impact on Organizational Processes

The participants in our study explained how the simulation helped them to think about how their organization could incorporate more gender-sensitive practices, policies, and privacy considerations of whom the gendered information is shared with.

##### Gender-Sensitive Practices and Policies

Clinicians considered how intake forms were often binary (ie, male or female) with no other options for people who are nonbinary. For example, a clinician described,

The simulation highlighted the importance of reflecting on our organization processes regarding gender (e.g. how do we ask clients to report preferred pronouns, etc.)…The simulation did make me reflect that we don't typically ask who we can share information on preferred pronouns with so this is something for our program to consider moving forward.

#8, Occupational Therapist

##### Privacy

Other clinicians contemplated privacy issues regarding how the gender identity information would be shared, which is particularly concerning when working with youth who may not have discussed their gender identity with their parents. To illustrate, an occupational therapist said,

It's helpful to remember to ask the client who they want to share this information with.

#10, Occupational Therapist

## Discussion

### Principal Findings

Our study addressed an important gap in the literature by exploring a preliminary evaluation of an educational simulation for health care and service providers on gender-sensitive approaches within the context of providing vocational rehabilitation for youth with disabilities. Offering training on this topic could assist with addressing health and social inequalities and optimizing patient outcomes [9]. Educational simulations, such as this one, have the potential to support clinicians with recognizing their own cultural biases, prejudices, and assumptions in a safe environment without risking stigmatization of a patient in a clinical setting [32].

The results of our study underscored the relevance and authenticity of the simulation content, which is an important finding for our pilot study because authenticity is an essential component of simulation and standardized patient methodology [48]. Our findings highlighted that our educational simulation has the potential to help inform personal knowledge or understanding of the topic and confidence in professional or daily practice activities. However, it is important to emphasize that further work that is tested with larger sample sizes is needed to examine the actual rather than perceived impact. The qualitative findings from our open-ended questions similarly highlighted the perceived role of the simulation for clinical practice, including gender-sensitive language and communication, and building rapport with patients. Our pilot findings suggest that the simulation has the potential to be a helpful tool to equip health care providers to recognize their own biases and use appropriate language. Other research also shows that simulations could help to reduce discomfort and discrimination in health care for gender minority patients by equipping clinicians with sensitive and inclusive communication tools in a risk-free environment where they can learn to provide care to a vulnerability population [32].

Our findings align with principles of patient-centered and culturally sensitive care models that place emphasis on self-awareness and sensitivity to one’s own values, biases, and power differences while also considering patients’ values and priorities [49]. Previous research shows that health care providers could benefit from an ongoing reflective practice and awareness of their values, assumptions, and beliefs [50]. In addition, enhancing knowledge and learning opportunities on gender-sensitive approaches could help to alleviate clinicians’ fear of making mistakes, unintentionally causing harm, or creating unsafe space [51]. Other research shows how effective communication serves an essential role in ongoing patient-clinician relationships and patient satisfaction [5]. Indeed, effective gender-sensitive approaches and related communication could assist health care providers to better understand patients’ needs and priorities, which could help them to tailor their recommendations [5].

Our qualitative findings highlighted how participants reflected on the potential role that gender-sensitive care could play in organizational processes. It was encouraging to see that participants were aware of how gender operates at an institutional level and acknowledged how power dynamics at a leadership level could influence practice at the everyday individual level (eg, forms that are binary male or female). Other research on gender-sensitive approaches using simulation training in nursing found that it helped nurses to reflect on the importance of inclusionary hospital policies to protect the rights and dignity of people who are trans [32]. Previous studies indicate that a critical element of creating a more inclusive and welcoming environment for the LGBTQSi+ community involves adopting antidiscrimination policies and ensuring that they feel supported [52]. It is essential that senior leadership of health care organizations assist with improving the implementation and uptake of gender sensitivity training in order for it to achieve optimal benefits [3,53]. Indeed, it is important to understand that gender is part of a larger sociopolitical and cultural context and that health care organizations are often gendered [3], a view that aligns with the World Health Organization’s report on the social determinants of health [1]. Thus, it is critical to consider how the intersection of multiple identities (eg, sexual orientation, socioeconomic status, age, ethnicity, etc) shapes patients’ health care needs and, in turn, clinicians’ responses to them [3,54].

### Limitations

Caution should be used in interpreting the findings because the majority of the participants in our study self-identified as women. Nevertheless, such a gender-unbalanced sample is a reflection of the gender composition of health care providers within pediatric rehabilitation [55]. Future research should consider a purposive sampling of nonbinary or gender fluid clinicians. Additionally, it would be worthwhile for future simulations to incorporate a variety of different gender and disability identities (eg, physical and sensory disabilities). Further research should also explore the impact of sociodemographic characteristics on perceived outcomes of the tool. Second, this pilot evaluation involved a small qualitative sample drawn from one city, and caution should be used in generalizing the findings. We did not reach our target sample size in large part because of restrictions with conducting research in a health care setting during the COVID-19 pandemic. It is also important to note that 2 participants did not complete all of the questions, and there were some missing data. Therefore, it would be worthwhile to evaluate the tool with a larger sample while including longer-term outcomes (ie, intention to change practice or behaviors and actual changes implemented) and a control group. Finally, viewing educational simulations individually may not result in the same impact when it is viewed as a group. Future studies should explore the impact of different formats and educational settings.

### Conclusions

This pilot study provided preliminary evidence of the usefulness of a gender-sensitive educational simulation. Our results highlighted that the content of the simulation was comprehensive and relevant. Our pilot findings showed that participants perceived that the simulation was helpful for understanding gender-sensitive care, their approach, and their perceived confidence in their daily practice. The qualitative findings emphasized the importance of gender-sensitive language and communication, and building rapport with patients. Further studies should explore the longer-term impact of this educational simulation on clinical practice and behaviors.

## Appendix

Multimedia Appendix 1 Table overview of themes.

## Abbreviations

LGBTQ2si+: lesbian, gay, bisexual, trans, or queer two-spirited
REDCap: Research Electronic Data Capture

## Data Availability

The data sets generated during the current study are not publicly available due to restrictions from our institutional research ethics board. However, an anonymized and deidentified version can be made available from the corresponding author (with approval from our research ethics board) on reasonable request.
