# Supplementary material for: A Web-Based Gender-Sensitive Educational Simulation on Vocational Rehabilitation for Service Providers Working With Youth With Disabilities: Pilot Evaluation
Source: JMIR Form Res. 2023 Mar 24;7:e38540. doi: 10.2196/38540 (PMC10131866; doi:10.2196/38540)
Supplement: Multimedia Appendix 1 [file formative_v7i1e38540_app1.docx]

**Multimedia appendix 1. Table overview of themes and sub-themes**

| **Themes** | **Sub-themes** | **Exemplar quotes** |
| --- | --- | --- |
| Relevance of the simulation content and format | Relatable content | -“The video was extremely valuable…I found the simulations to be very useful and  informative. They were incredibly impactful and made the information tangible”  (#2, Youth Facilitator).  -“The simulation was engaging” (#11, occupational therapist)  -“Seeing where education is needed, especially in workplaces not particularly familiar  with accommodations for those with disabilities…The tool was useful as it serves as  great education of how clients are treated/ gives us a glimpse of how they may feel  and helps us work towards betterment in a holistic way.” (#1, Occupational Therapy  Assistant) |
|  | Suggestions for improvement | -“I would have liked to have seen additional resources specific to employment best  Practices.” (#8, Occupational Therapist) |
| Perceived impact on clinical practice |  | -“The simulations provided the viewer with how to make improvements in their  approach, treatment, and interaction when dealing with persons living with  disabilities and in terms of gender sensitivity.” (#1, Occupational Therapy Assistant) |
|  | Gender-sensitive language and communication | -“I learned a lot through observing the clinician in conversation with the client. It  gave me a thorough understanding of the language to use when speaking with  clients.” (#12, Social Worker)  -“The simulations were helpful in seeing what to do and what not to do when  speaking about gender identity. It was also helpful in seeing how to navigate tough  conversations with clients related to gender identity.” (#10, Occupational Therapist)  -“It really highlighted for me to not make assumptions based on my own gender bias  (e.g. a patient's appearance, what may be listed in their chart, etc.) and the  importance of making sure our forms include more than ‘two boxes’ as referred to in  the video.” (#6, Occupational Therapist) |
|  | Building rapport with clients | -“I found the simulation to be very helpful. I will use this information when I am  facilitating conversations with clients and their families/caregivers in my clinical  role.” (#2, Youth Facilitator)  -“If a clinician does make a mistake then its important they apologize and  acknowledge that they will do their best to address the client appropriately. I think  it’s also beneficial that the clinician stated she may make an error and asked that the  client help her if this occurs…It's important to not just take the information from the  chart but to speak to the client before making any assumptions regarding gender  identity.” (#10, Occupational Therapist) |
| Perceived impact on organizational processes | Gender-sensitive practices and policies | -“In the past year we've updated our application form to include pronouns, so that the  youth can share theirs. Although this could change over time, I think it's important to  ask them in that initial meeting to ensure no assumptions are being made from the  start.” (#6, Occupational Therapist) |
|  | Privacy | -“It also raises important questions regarding the consumers choice of who they  would like to share this information with but it is also relevant for many aspects of  clients interface with the healthcare system.” (#3, Social Worker) |
